# 3D-Printed PLA Mechanical and Viscoelastic Behavior Dependence on the Nozzle Temperature and Printing Orientation

**DOI:** 10.3390/polym17070913

**Published:** 2025-03-28

**Authors:** Lykourgos C. Kontaxis, Dimos Zachos, Aliona Georgali-Fickel, Diana V. Portan, Stefanos P. Zaoutsos, George C. Papanicolaou

**Affiliations:** 1 Department of Mechanical Engineering and Aeronautics, University of Patras, 26504 Patras, Greece; aliona.georgali@yahoo.com (A.G.-F.); diana.portan@gmail.com (D.V.P.); gpapan@upatras.gr (G.C.P.); 2 Department of Energy Systems, University of Thessaly, 41500 Larisa, Greece; dimozach@uth.gr (D.Z.); szaoutsos@uth.gr (S.P.Z.)

**Keywords:** 3D-printing, fused deposition modeling (FDM), polylactic acid (PLA), dynamic mechanical analysis (DMA), differential scanning calorimetry (DSC), degree of crystallinity, mechanical properties, printing orientation, glass transition region (T_g_), nozzle temperature

## Abstract

The present study focuses on the mechanical and viscoelastic characterization of 3D-printed PLA, fabricated in three different printing orientations (0°, 45°, and 90°) and four different nozzle temperatures (210, 220, 230, and 240 °C). By employing a combination of static and dynamic mechanical analysis (DMA) testing, as well as differential scanning calorimetry (DSC) analysis, this work aims to investigate the relationship between processing parameters and the resulting properties of PLA. DSC results showed that higher nozzle temperatures enhance the degree of crystallinity, which in turn affects the mechanical and viscoelastic behavior of PLA. Regardless of the nozzle temperature, the flexural strength decreased as the printing orientation degrees increased. However, it was found that the higher the nozzle temperature, the higher the flexural strength for the same orientation, and the smaller the strength deviations per specimen. DMA results indicated that as the printing orientation increased, glass transition temperature (T_g_) values increased while storage modulus values decreased. At the same time, in both cases, by increasing nozzle temperature, an increase in T_g_ and a respective increase in storage modulus values is observed due to the increase in the degree of crystallinity.

## 1. Introduction

Additive manufacturing, particularly fused deposition modeling (FDM), has revolutionized the production of customized and complex structures across various industries. Among the materials commonly used in FDM, polylactic acid (PLA) stands out due to its biodegradability, ease of processing, and favorable mechanical properties [1]. PLA is among the main biodegradable plastics that are commonly used in the production of carrier bags, garbage sacks, food packaging, and other items [2]. Also, PLA is used in several applications, in direct and indirect contact with the human body. Due to the accessibility of the resources for its fabrication and biocompatibility, PLA is the most appropriate thermoplastic that is tolerated by both the human organism and the environment. Recent research focused on the application of PLA in food packaging. For this purpose, antifouling antibacterial polylactic acid (PLA)-based packaging has been developed, and its performance has been studied [3]. In the biomedical direction, biocompatible thermoplastics, such as PLA, are proposed for the coating of implantable metals [4]. In the complex conditions of the human body, PLA properties and their feedback to a relevant environment have to be very well understood. The role of PLA coatings on implants is to efficiently intermediate the adhesion between the natural tissue and the implant material, especially in cases where the nanostructured nature of the substrate restrains biorecognition and biointegration [5,6,7].

However, the mechanical and structural performance of 3D-printed PLA components is highly dependent on processing parameters, such as printing orientation and nozzle temperature [8,9]. These parameters influence critical aspects of the printed parts, including layer adhesion, crystallinity, and molecular alignment, ultimately determining the material’s overall performance. Understanding the effects of these parameters is essential for optimizing the fabrication process and expanding the applicability of 3D-printed PLA in engineering and biomedical applications [10,11,12,13,14]. Printing orientation plays a significant role in determining the mechanical anisotropy of 3D-printed parts, as it affects the alignment of polymer chains and the distribution of stresses within the material [15,16,17,18]. Afrose et al. [19] tested FDM-printed Polylactic Acid (PLA) parts in three specific orientations, namely X, Y, and 45°. The PLA-X specimens were found to have the highest tensile stress, while, under cyclic loading conditions, PLA-45 showed the highest fatigue life compared to the other two orientations. Vindedze et al. [17] studied the tensile characteristics of Ultem 9085 (a blend of polyetherimide and polycarbonate) for samples printed in three different orientations (X, Y, and Z). The results revealed that mechanical properties, such as elastic modulus and tensile strength, significantly differed from the Z printing orientation, particularly in the X and Y printing layer orientations, with the Y orientations being optimal. More recently, Syaefudin et al. studied the effect of three printing orientations (0°, 45°, and 90°) on the tensile strength of 3D-printed ABS and PLA materials [20]. The results showed once more that the orientation influences the tensile strength of the ABS and PLA samples. The change in orientation from 0° to 90° caused a significant decrease in tensile strength of 44.3% of ABS and 52.8% of PLA materials, showcasing the importance of the printing orientation.

Similarly, nozzle temperature influences interlayer bonding, thermal history, and the degree of crystallinity, all of which contribute to the final mechanical properties [21,22]. Ansari et al., as well as Alafaghani et al., documented an increase in tensile strength with the increase in nozzle temperature, in conjunction with the optimal printing speed [23,24]. Similarly, Rivera-López et al. [25] found an increase in both tensile and flexural strength with the increase in nozzle temperature. On the contrary, de Freitas et al. [26] found a decrease in tensile strength with the increase in nozzle temperature, while using a low-end printer, signifying the hardware importance in 3D printing. Kuznetsov et al. [27] specifically concluded that the design and build quality of FDM 3D printers can have both a quantitative and qualitative effect on the printed samples’ strength, and that researchers should be careful to correlate the results of their experiments to the specific class of the FDM devices on which the specimens were manufactured. While numerous studies have explored the mechanical behavior of 3D-printed PLA, a comprehensive investigation into the combined effects of printing orientation and nozzle temperature on its structural and mechanical properties remains limited. Such an understanding is crucial for tailoring the material’s performance to meet specific application requirements.

The present study focuses on the mechanical and viscoelastic characterization of 3D-printed PLA plates fabricated in three different printing orientations (0°, 45°, and 90°) and four different nozzle temperatures (210, 220, 230, and 240 °C). By employing a combination of static and dynamic mechanical analysis (DMA) testing, as well as differential scanning calorimetry (DSC) analysis, this work aims to investigate the relationship between processing parameters and the resulting properties of PLA. The findings provide valuable insights into the optimization of FDM parameters, enabling the production of PLA components with enhanced mechanical performance and tailored properties for diverse applications.

## 2. Materials and Methods

### 2.1. Materials and 3D-Printing Settings

**Figure 1:**
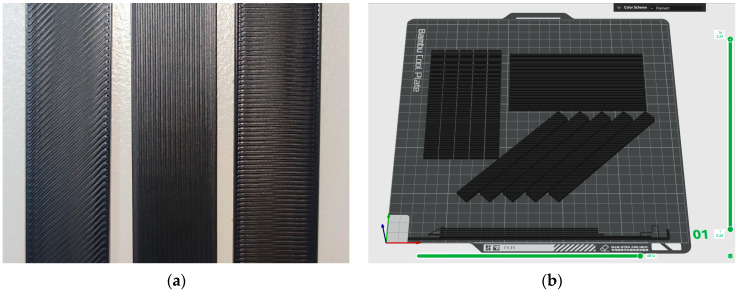
Flexural specimens in accordance with ASTM D0790 (**a**) Close-up on the three different orientations 45°, 0°, and 90° (left to right) (**b**) Three-dimensional printing setup using Bambu Studio software.

Polylactic acid (PLA) filament with a diameter of 1.75, named PLA Basic, was purchased from Bambu Lab (Shenzhen, China). The material had a density of 1.24 g∙cm^−3^, a melting temperature of 160 °C, a flexural modulus of 2.75 ± 0.16 GPa, and a flexural strength of 76 ± 5 MPa, according to the manufacturer. A Bambu Lab X1 Carbon desktop 3D printer (Shenzhen, China) based on FDM has been used to manufacture all the specimens. Three different printing orientations of 0°, 45°, and 90° (Figure 1a), and four different nozzle temperatures (210, 220, 230, and 240 °C) were applied. The software Bambu Studio v1.10.2.76 was used, and the key printing parameters chosen are presented in Table 1.

### 2.2. Differential Scanning Calorimetry

Thermal characterization of the compounds was conducted by means of DSC measurements, via a TA Q200 DSC device (New Castle, DE, USA), in the temperature range of 30–250 °C, operating at 10 °Cmin^−1^ in endothermic mode. Experimental tests were conducted in a nitrogen atmosphere using about 4.5–5.3 mg samples sealed in aluminum pans, while an empty aluminum pan served as a reference. The data obtained from the DSC measurements (enthalpy changes ΔH, characteristic transition, and peak temperatures) were analyzed using TA Universal Analysis v4.5A software. During thermal scans, cold crystallization enthalpies, ΔH*_cc_*, melting temperature, T_m_, and melting enthalpies, ΔH*_m_*_,_ were recorded. The degree of crystallinity, *Χ_c_*, of the different samples was calculated by the following equation:

(1) $$X_{c}(\%)=\frac{\Delta H_{m}-\Delta H_{cc}}{\Delta H_{m{}^{\circ}}}\cdot 100.$$

ΔH*_m°_* is a reference value that represents the heat of crystallization for a 100% crystalline polymer (for a 100% crystalline PLA, ΔH*_m°_* = 93 J/g) [22,28,29].

### 2.3. Quasi-Static Mechanical Test

Quasi-static flexural properties of the materials manufactured were determined under three-point bending loading, in accordance with ASTM D0790 [30], and were tested in a universal testing machine, INSTRON 3382 (High Wycombe, UK), with a displacement rate of 2 mm/min and a span length of 64mm. All specimens used for the flexural tests had dimensions 100 × 12.7 × 3.2 mm^3^ and were printed as seen in Figure 1b. Five or more specimens per filler weight fraction were tested to ensure the repeatability of results. Flexural stress, σ, and flexural strain, ε, values were calculated according to Equations (2) and (3), respectively.

(2) $$\sigma =\frac{3\cdot Force\cdot L_{s}}{2\cdot w\cdot d^{2}},$$

(3) $$\varepsilon =\frac{6\cdot displacement\cdot d}{L_{s}},$$

where *L_s_* is the span length, *w* is the width, and *d* is the depth of the specimen.

### 2.4. Dynamic Mechanical Analysis

DMA measurements were executed on PLA rectangular 3D-printed specimens with mean dimensions of 60 × 12 × 3.5 mm^3^ in order to study the effect of the manufacturing parameters on both the glass transition temperature (T_g_) and the storage modulus (E′) values of the PLA specimens. The specimens were characterized under a three-point bending mode, utilizing a dual cantilever clamp, as a function of temperature (30–150 °C at 1 Hz) using a Q800 TA-instruments DMA device (New Castle, DE, USA). The specimens had a span length of 50mm and an oscillation amplitude of 20 μm. Five or more specimens per case were tested to ensure the repeatability of results.

## 3. Results and Discussion

### 3.1. DSC Analysis of PLA

**Figure 2:**
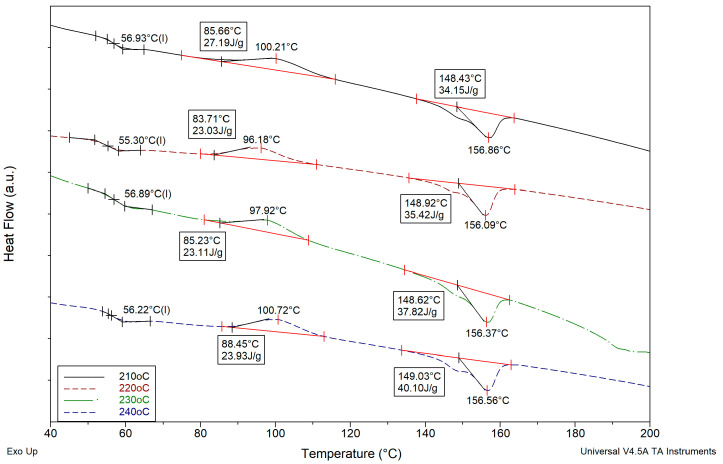
DSC thermographs for PLA specimens manufactured using four different nozzle temperatures (210, 220, 230, and 240 °C).

In Figure 2, DSC thermographs corresponding to the four different nozzle temperatures used for the manufacturing of PLA specimens are shown.

**Figure 3:**
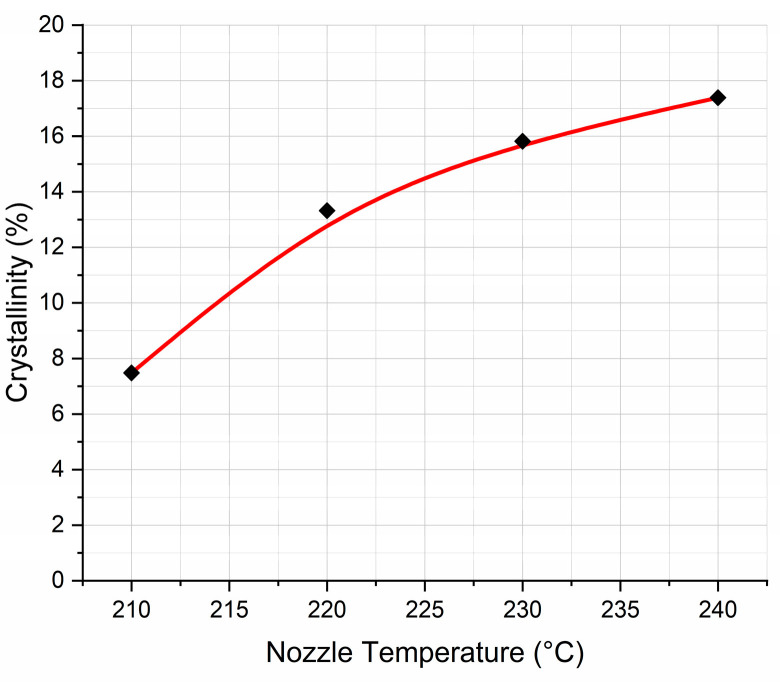
Degree of crystallinity against nozzle temperature.

From these thermographs, detailed information concerning T_g_ values, ΔH enthalpies, and T_m_ values is calculated using TA Universal Analysis v4.5A software. Also, from Equation (1), using the above data, exact values for the degree of crystallinity were calculated and tabulated in Table 2 and plotted as a function of nozzle temperature in Figure 3.

From the data tabulated in Table 2, small variations in the T_g_ and T_m_ values are observed. However, as expected, a noticeable increase in the degree of crystallinity with increasing nozzle temperature is clear. Thermoplastics, during their preparation stage, may develop crystallites. The formation of crystallites occurs during the cooling phase. The faster the cooling rate, the smaller the crystallites are. Conversely, the slower the cooling rate, the larger the crystals that will grow. The nozzle temperature has a significant impact on the mechanical properties of the 3D-printed object [31,32]. Specifically, the nozzle temperature influences the rheology of the polymer melts passing through the nozzle, as well as the crystallinity of the 3D-printed structure, which are the major characteristics influencing the mechanical properties of the 3D-printed structure. Furthermore, the nozzle temperature might alter the properties of a fully 3D-printed layer while printing the subsequent layers. At temperatures exceeding the melting temperature, T_m_, there is a significant amount of energy in the system, and the segmental motion of the polymer chains is too fast for stable nuclei to form and crystalline development to happen [33,34]. However, as the temperature drops below T_m_, melt viscosity increases, increasing the possibility of nucleation and the formation of a microstructure with a few but very large crystals. As the temperature drops further and the viscosity increases, the rate of crystallization reaches a maximum where conditions favor both nucleation and development of crystallites [33]. When approaching T_g_, the system has substantially less energy, and the molecular mobility of the polymer chains is greatly diminished. There is a much lower nucleation barrier opposing the creation of stable nuclei in such a viscous condition; however, the transport of the chains to the crystal growth front is hampered due to reduced molecular mobility [34]. This results in a microstructure with a greater number of crystallites but a much smaller size. Below T_g_, the polymer chains are effectively ’frozen’ in situ, and no further crystallization can occur [35,36]. Thus, for a fixed plate temperature and printing speed, higher nozzle temperatures lead to slower cooling, thereby enhancing crystallization, as depicted in Figure 3.

### 3.2. Quasi-Static Flexural Properties

**Figure 4:**
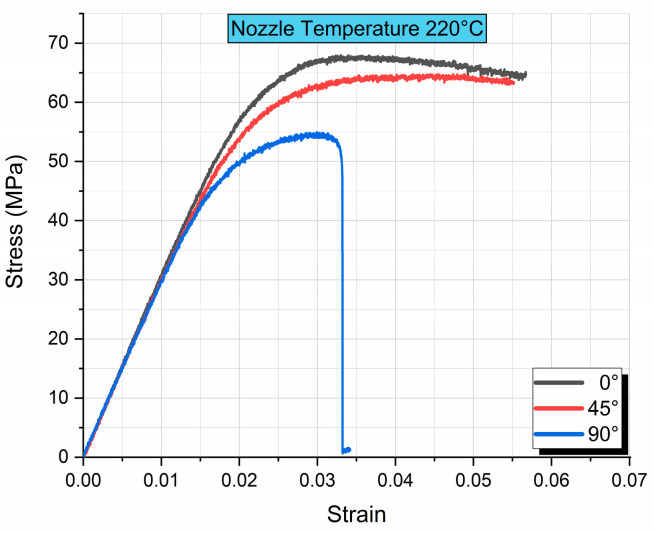
Comparison of stress–strain curves between different orientations for a nozzle temperature of 220 °C.

Flexural modulus was calculated from the slope of the stress–strain curve, while flexural strength was derived by measuring the maximum stress observed by each specimen. In Figure 4, stress–strain curves are plotted and presented. Figure 4 presents a comparison of the stress–strain curves for the three different printing orientations. In accordance with ASTM D0790, the tests were terminated “when the maximum strain in the outer surface of the test specimen has reached 0.05 mm/mm [in./in.] or at break if break occurs prior to reaching the maximum strain”, which corresponds to roughly 10 mm deflection or 0.05 strain, depending on the exact depth of each specimen.

As expected and showcased in previous research [20,23,25,37], the specimens with 0° orientation exhibit the highest flexural strength and, overall, the best mechanical behavior, while the 45° orientation exhibits a similar curve but with lower flexural strength, as seen in Figure 4. Finally, the 90° orientation displays the lowest flexural strength as expected, since the loading is off-axis of the printing orientation. Especially on the “top” face of the specimen that experiences tensile stresses, the 90° orientation means that the layer lines are perpendicular to the loading direction, and the stresses developed depend on the inter-layer bonding strength, rather than the layer lines themselves, as it is the case of the 0° orientation specimens. This dependency of the 90° orientation in the inter-layer bonding strength also explains the lower repeatability of results, as shown in Table 3. In Table 3, flexural modulus and flexural strength values, along with their corresponding coefficients of variation, are tabulated and presented.

**Figure 5:**
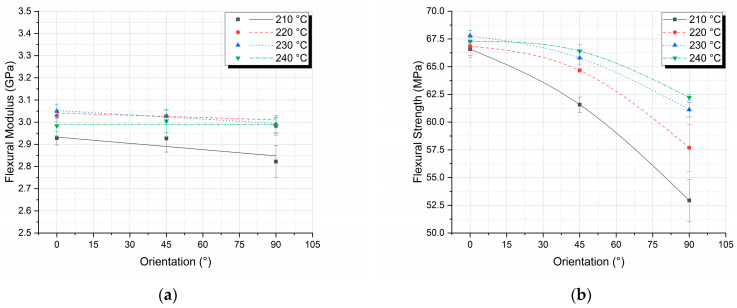
Flexural properties versus printing orientation for different nozzle temperatures: (**a**) flexural modulus; (**b**) flexural strength.

As observed in Table 3, the 90° orientation specimens exhibit the largest coefficient of variation differences, while the 0° and 45° specimens are practically identical, with the largest variation being on the order of 1.5%. This is attributed to the poor adhesion and the discontinuities created between the layer lines during the manufacturing process, greatly reducing the specimen’s flexural strength. However, it is worth noting that, especially nowadays, with the creation of much more precise 3D printers, the specimens manufactured have satisfactory repeatability, displaying a coefficient of variation of less than 4% in the worst cases. Finally, when it comes to the flexural modulus, a small variation was found; nevertheless, an existing one that cannot be distinguished by just examining Figure 4, but only after calculating the slopes of the stress–strain curves. In Figure 5, flexural modulus (Figure 5a) and flexural strength (Figure 5b) are presented in relation to printing orientation for the four different nozzle temperatures. 

The flexural modulus decreases as the orientation changes from 0° to 90°, even if ever so slightly. The largest difference between flexural moduli occurs at the 90° orientation, between specimens extruded with 240 °C and 210 °C nozzle temperatures, and is on the order of 6% (Figure 5a). Although this is not a very large variation between specimens, the low standard deviation (i.e., high repeatability of results) between specimens indicates that this is a measurable and quantifiable phenomenon. Concluding, in Figure 5a, a decrease in flexural modulus is observed as the printing orientation degrees increase. This decrease is more pronounced for the lowest nozzle temperature of 210 °C and is attributed to the poor adhesion between the layer lines due to the lower nozzle temperature. On the contrary, the curves for the remaining nozzle temperatures above 220 °C, which is the recommended nozzle temperature by the manufacturer, are grouped, indicating that the layer lines have bonded sufficiently.

**Figure 6:**
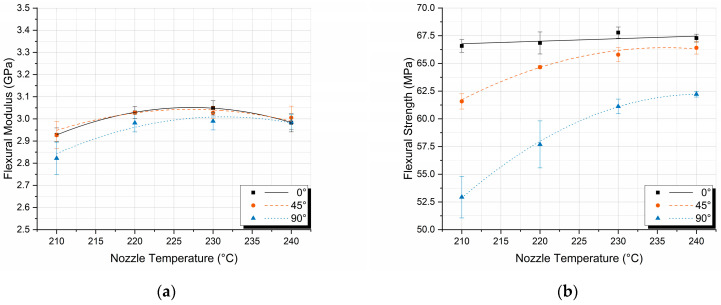
Flexural properties versus nozzle temperature for different printing orientations: (**a**) flexural modulus; (**b**) flexural strength.

Concerning the flexural strength, as the printing orientation degrees increase, the flexural strength decreases in all cases of nozzle temperatures (Figure 5b). This outcome was anticipated and is well documented [20,23,25,37], as previously discussed, due to the 90° orientation, where the layer lines are perpendicular to the applied load. In this configuration, the load-bearing capacity is primarily determined by the inter-layer bonding strength between the layers rather than the properties of the layer lines themselves, as observed in specimens oriented at 0°. Finally, in Figure 5b, it is observed that as the nozzle temperature increases, the rate at which the flexural strength decreases also decreases. To gain a deeper understanding of these phenomena, additional cross-plots were constructed. In Figure 6, flexural modulus (Figure 6a) and flexural strength (Figure 6b) are presented in relation to nozzle temperature for the three different printing orientations. 

The first conclusion that can be extracted from Figure 6a is that the nozzle temperature of 210 °C results in the lowest flexural moduli for all printing orientations. Once reaching the recommended nozzle temperature of 220 °C, the modulus remains almost constant for the individual printing orientations, with the largest difference observed being on the order of 0.07 GPa, or 2.34%, for the case of the printing orientation of 0° and the temperatures of 230 °C and 240 °C. Nevertheless, deviations between the printing orientations are observed, especially for the 90° orientation; however, they are not as severe as the ones observed in Figure 5a, leading to the conclusion that the nozzle temperature affects the flexural modulus more than the orientation of the layer lines printing. This is attributed to the fact that thermoplastics can develop crystals during the cooling phase, with the crystal size being influenced by the cooling rate; faster cooling results in smaller to no crystals, while slower cooling leads to larger crystals. The nozzle temperature plays a crucial role in the mechanical properties of 3D-printed objects by affecting the polymer melt’s rheology and the crystallinity of the printed structure. As confirmed by the DSC experiments, for a fixed plate temperature and printing speed, higher nozzle temperatures lead to slower cooling, enhancing crystallization and modifying the mechanical properties, like increasing the flexural modulus.

On the contrary, the analysis of both Figure 5b and Figure 6b demonstrates that the printing orientation has a greater impact on the flexural strength of the specimens than the nozzle temperature. The flexural strength for 0° printing orientation remains the highest as expected, but also remains constant and independent of the nozzle temperature. This is attributed to the orientation of the layer lines matching the tensile loads. This factor dominates the temperature printing parameter, leading to a linear behavior with a slope approaching zero (0.02). For the printing orientations of 45° and 90°, however, the higher the temperature, the higher the flexural strength due to better inter-layer bonding, as explained previously [24,38,39]. Finally, it is observed, as discussed in Table 3, that the deviations in the 90° specimens are considerably larger; however, these deviations decrease as the nozzle temperature increases. This observation led to the conclusion that for the off-axis orientations, the higher the nozzle temperature, the higher the flexural strength, and the smaller the strength deviations per specimen.

### 3.3. DMA Results

**Figure 7:**
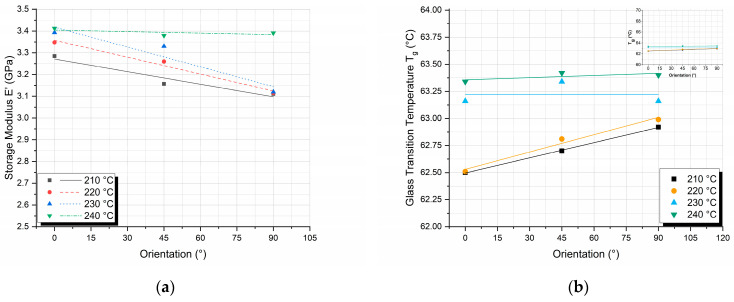
Effect of printing orientation on the (**a**) storage modulus E′ and (**b**) glass transition temperature T_g_ values for different nozzle temperatures.

Dynamic mechanical analysis was performed to investigate the effect of nozzle temperature and printing orientation on the storage modulus (E′) and T_g_. The variations in E′ (Figure 7a) and T_g_ (Figure 7b) as a function of printing orientations for different nozzle temperatures are presented in Figure 7. The E′ values were measured at 30 °C under a frequency of 1 Hz, while the T_g_ values were determined from the peaks of the tanδ curves.

The E′ exhibits a decreasing trend as the printing orientation changes from 0° to 90°, consistent with the behavior observed in the flexural modulus. However, in the case of dynamic results, the difference in moduli is more pronounced. The largest difference between storage moduli occurs at 90° orientation as well, between specimens printed with 210 °C and 240 °C nozzle temperatures, and is on the order of 9% (Figure 7a). However, in contrast with the flexural modulus, where specimens for nozzle temperatures above 220 °C exhibited similar behavior and were less influenced by the printing orientation; the E′ for nozzle temperatures below 240 °C decreases with increasing printing orientation. This leads to the formation of a separate grouping in the DMA results and is attributed to the dynamic nature of the experiment, concluding that under dynamic loading, sufficient bonding between layer lines is achieved only at the highest nozzle temperature of 240 °C.

In contrast, the glass transition temperature, as derived by the DMA experiments, demonstrates a slight increase with the increase in the printing degree of orientation, particularly for specimens printed at temperatures of 210 °C and 220 °C. However, this observed increase is minimal, around 0.5 °C, which falls within the margin of experimental error. Therefore, the variation in T_g_ with respect to printing orientation is not deemed statistically significant under these conditions. This observation is more clearly illustrated in the inset graph of Figure 5b (located in the upper right corner), where the *y*-axis scale has been enlarged to highlight the minimal changes in T_g_ values.

**Figure 8:**
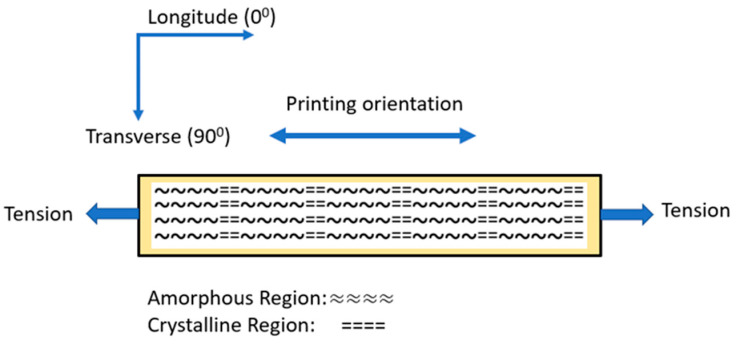
Effect of printing orientation on the crystallite’s orientation and PLA plate anisotropy.

The printing orientation affects the mechanical properties of the FDM 3D-printed parts because it affects the anisotropy of the printed part (Figure 8) [40].

By increasing the nozzle temperature, there is a resulting increase in the degree of crystallinity. On the other hand, printing orientation introduces anisotropy in the printed PLA specimens. Thus, the properties of the printed PLA specimens in the printing longitudinal direction are mainly affected by the crystals developed within the PLA structure, while the respective properties in the transverse direction are mainly dominated by the amorphous phase properties. In conclusion, it can be observed that as the printing orientation is increased, T_g_ values increase while storage modulus values decrease; at the same time, in both cases, by increasing nozzle temperature, an increase in T_g_ and a respective increase in storage modulus values is observed due to the increase in the degree of crystallinity. In order to evaluate the effect of nozzle temperature on E′ and T_g_ values, additional cross-plots were constructed.

**Figure 9:**
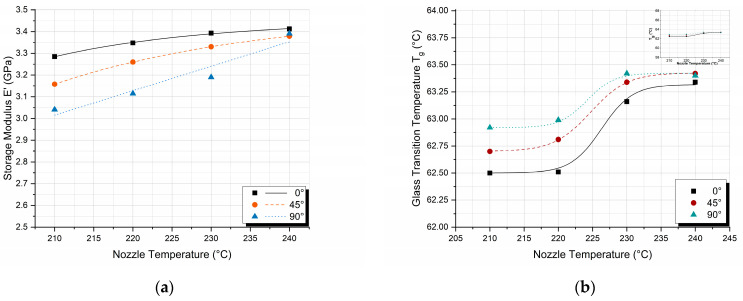
Effect of nozzle temperature on the (**a**) storage modulus E′ and (**b**) glass transition temperature T_g_ values for different printing orientations.

In Figure 9, the variations in E′ (Figure 9a) and T_g_ (Figure 9b) as a function of nozzle temperatures for different printing orientations. It is noticeable from both Figure 9a,b that the observed differences are again too small; however, since they are repeatable during the experimental testing, in the following paragraph, a plausible explanation is given.

Crystals perturb the amorphous phase and diminish its segmental mobility, giving the amorphous phase a T_g_ distribution. Amorphous regions adjacent to the crystal surface will experience the greatest degree of restriction, with the effect decreasing as one advances through the interface and reaches the amorphous phase unconstrained by crystallites, having properties equivalent to those of the bulk amorphous material [41,42]. As a result of the above-mentioned mechanisms, both T_g_ and storage modulus values are expected to increase with increasing nozzle temperature, as shown in Figure 9.

## 4. Conclusions

In the present study, the effects of manufacturing parameters, such as printing orientation and nozzle temperature, on the mechanical and viscoelastic behavior of 3D-printed PLA specimens were investigated. The following main conclusions were drawn:

- From DSC results it is concluded that for a fixed plate temperature and printing speed, higher nozzle temperatures lead to slower cooling, enhancing the degree of crystallinity.
- The nozzle temperature of 210 °C results in the lowest flexural moduli for all printing orientations. However, upon reaching the recommended nozzle temperature of 220 °C, the modulus remains almost constant for all the individual printing orientations.
- Concerning the flexural strength, as the printing orientation degrees increase, the flexural strength decreases in all cases of nozzle temperatures. For the off-axis orientations, the higher the nozzle temperature, the higher the flexural strength, and the smaller the strength deviations per specimen, while for 0° orientation, the flexural strength remains constant with nozzle temperature.
- It was observed that as the printing orientation is increased, T_g_ values increase while storage modulus values decrease; at the same time, in both cases, by increasing nozzle temperature, an increase in T_g_ and a respective increase in storage modulus values is observed due to the increase in the degree of crystallinity.

## Figures and Tables

**Table 1:** Key printing parameters selected.

Parameter	Value
Layer Height	0.2 mm
Line Width	0.4 mm
Wall loops	1
Infill Pattern	Aligned Rectilinear
Infill Density	100%
Top/Bottom Layers	0
Recommended nozzle temperature	220 °C

**Table 2:** Data derived from the DSC experiments.

Nozzle Temperature (°C)	T_g_ (°C)	T_m_ (°C)	ΔH*_cc_* (J/g)	ΔH*_m_* (J/g)	Degree of Crystallinity (%)
210	56.93	156.86	27.19	34.15	7.48
220	55.30	156.09	23.03	35.42	13.32
230	56.89	156.37	23.11	37.82	15.82
240	56.22	156.56	23.93	40.10	17.39

**Table 3:** Quasi-static flexural modulus and flexural strength values, accompanied by coefficients of variation.

Nozzle Temperature (°C)	Orientation	Flexural Modulus (GPa)	Coefficients of Variation (%)	Flexural Strength (MPa)	Coefficients of Variation (%)
210	0°	2.92	1.0	66.58	0.85
	45°	2.93	2.10	61.57	1.12
	90°	2.82	2.59	52.94	3.55
220	0°	3.03	0.90	66.85	1.49
	45°	3.03	0.24	64.67	0.24
	90°	2.98	1.37	57.69	3.67
230	0°	3.05	1.05	67.78	0.74
	45°	3.03	0.89	65.79	0.97
	90°	2.99	1.33	61.12	1.09
240	0°	2.98	1.39	67.29	0.53
	45°	3.01	1.75	66.41	0.85
	90°	2.99	1.10	62.22	0.41

## Data Availability

The dataset is available on request from the authors. (Data are contained within the article; raw data will be available upon request to the corresponding author).

## Abbreviations

The following abbreviations are used in this manuscript:

FDM	Fused deposition modeling
PLA	Polylactic acid
DSC	Differential scanning calorimetry
DMA	Dynamic mechanical analysis
T_g_	Glass transition temperature
E′	Storage modulus
